# Biological Activities of Alkaloids: From Toxicology to Pharmacology

**DOI:** 10.3390/toxins12040210

**Published:** 2020-03-26

**Authors:** Zbigniew Adamski, Linda L. Blythe, Luigi Milella, Sabino A. Bufo

**Affiliations:** 1 Department of Animal Physiology and Development/Electron and Confocal Microscope Laboratory, Faculty of Biology, Adam Mickiewicz University, 61-614 Poznań, Poland; 2 Department of Veterinary Medicine, Oregon State University, Corvallis, 97331 OR, USA; Linda.Blythe@oregonstate.edu; 3 Department of Science, University of Basilicata, 85100 Potenza, Italy; luigi.milella@unibas.it

Plants produce many secondary metabolites, which reveal biological activity. Among them, alkaloids demonstrate a broad spectrum of activities. In nature, they not only are produced against herbivores but also reduce bacterial or fungal infestation. Therefore, they are substances that possess high potential in medicine, plant protection, veterinary, or toxicology. Hence, the research on these substances and their properties develops intensively in many areas. The studies describing the physiological, pharmacological, and toxicological activity of alkaloids for different organisms belonging to every kingdom are of very wide interest. Both pure alkaloids and extracts are studied, and their activities are compared. In the Special Issue “Biological Activities of Alkaloids: From Toxicology to Pharmacology", 15 manuscripts describing ecological, biological, pharmacological, and toxicological effects as well as structural and analytical aspects of plant alkaloids, their mode of action, and possible application in veterinary, medicine, and plan protection were collected. The subjects focused on two main areas of interest, the structure/activity nexus and the application of alkaloids against pathogens.

Although the number of research articles on alkaloids increases, our knowledge of them is still far from completeness. This is due to the very high number of alkaloids produced by many different organisms, mostly plants, diffused all over the world. Therefore, the identification, characterization, and quantification of alkaloids present in plant species and their parts is very important and brings interesting data [1,2]. The spectrum of alkaloids’ activity is also very wide. Among them, there are substances showing antiviral, antibacterial, anti-inflammatory, and anticancer properties. Thus, many studies deal with curative aspects of alkaloids and their mode of action. *Mahonia aquifolia*, *Meconopsis cambrica*, *Corydalis lutea*, *Dicentra spectabilis*, *Fumaria officinalis*, and *Macleaya cordata* plant extracts showed cytotoxic activity against the tested human squamous carcinoma and adenocarcinoma cells [1]. The extracts obtained from the stem bark of *Rutidea parviflora* (*R. parviflora*) revealed significant cytotoxic activity against ovarian cancer. In this study, palmatine from the stem bark of *R. parviflora* was more toxic for human ovarian cancer cells than for human ovarian noncancerous cells [3]. Such basic studies are necessary and determine a very important point for the development of new anticancer drugs and therapies. In addition, sanguinarine and berberine, the isoquinoline alkaloids, revealed cytotoxic activity against hematopoietic cancer cell lines and induced apoptosis in the tested cell lines [4]. Curine—a bisbenzylisoquinoline alkaloid—was proven to modulate inflammatory effects in mice, due to the inhibition of macrophage activation and neutrophil recruitment, the inhibition of the production of cytokines and the decreased level of nitric oxide. The effects may be probably linked to the decreased level of nitric oxide and induced possibly by negatively modulating a Ca^2+^ influx [5]. The regulatory mode of the action of alkaloids refers also to other mechanisms within cellular membranes. Lindoldhamine (a bisbenzylisoquinoline alkaloid) was shown as a novel antagonist of acid-sensing ion channels (ASICs). Lindoldhamine significantly inhibited the ASIC1a channel’s response to physiologically relevant stimuli [6]. This observation is especially important, since only some molecules were described as modulators of ASIC1. That opens a new research area about bisbenzylisoquinoline alkaloids as important molecules in neurobiology. On the other hand, dehydrocrenatidine, a β-carboline alkaloid, suppresses voltage-gated sodium channels and leads to decreased allodynia. The alkaloid is the main component of *Picrasma quassioides*—a plant used in medicine, since it reveals antiviral activity, which is also known as an anti-inflammatory and analgesic agent. The research of Zhao and co-workers [7] brought important data on the mode of the action of this alkaloid.

Unfortunately, not all gold glitters: the consumption of some alkaloids may lead to toxic effects. Among them, there is arecoline, an alkaloid found for example in betel nuts. Overconsumption may lead to cancerogenesis and tumor formation. The mechanism of this effect is not fully known. Chang and co-workers described important aspects of the cancerogenic activity of arecoline [8]. The authors postulated that the mechanism uses a muscarinic acetylcholine receptor and the pathway that is triggered by the activation of this receptor. The authors described the effects of arecoline on cell migration and actin organization. The studies of that type may appear to be very important from the cytotoxicological, pharmacological, and clinical points of view.

Not only are cancer cells susceptible to alkaloids. The antiviral and antibacterial activity of alkaloids has already been described. This area of research appears to be important especially in the light of increasing the resistance of pathogenic bacteria to antibiotics. Casciaro and his co-workers presented an interesting study showing that nigritanine, an alkaloid obtained from *Strychnos nigritana*—a flowering plant that belongs to the family of Loganiaceae - possess high antibacterial activity against *Staphylococcus aureus* (*S. aureus*)*,* which is recognised to be one of the most important pathogenic bacteria diffused worldwide [9]. What appeared extremely important is the tested alkaloid did not reveal significant toxicity for mammalian red blood cells and human keratinocytes. The authors compared also the monomer/dimer structure–antibacterial activity relationship, which brought important information on the mechanism of activity against *S. aureus*. The research presented by Zielińska and her colleagues [10] included them in the same area of research. The authors showed a range of research on the presence of alkaloids in organs of *Chelidonium majus* and combined these observations with the activity of extracts and single metabolites against certain microorganisms: *S. aureus, Pseudomonas aeruginosa, Klebsiella pneumonia*, *Escherichia coli*, and *Candida albicans*. The results are in tune with the abovementioned research of Casciaro et al. [9] due to the described overall lower toxicity against eukaryotic cells (fibroblasts) than against microorganisms.

However, there are alkaloids that reveal toxic activity against animals. This seems obvious, since one of their main roles is to deter herbivory. Therefore, the wide range of alkaloids is described not only as substances with antimicrobial or anticancer agents but also as substances revealing insecticidal activity [11]. However, the nature of the toxic action of alkaloids on insects is still insufficiently described. In this issue, the effects of the activity of crude extracts obtained from *Solanum tuberosum*, *Solanum lycopersicum*, *Solanum nigrum* (Solanaceae), and *Armoracia rusticana* (Brassicaceae), as well as purified alkaloids, on the heart contractility of *Tenebrio molitor*—a pest of stored products—have been described [12]. In this research, chaconine was stated to be the most cardioactive substance among those tested. Apart from the information on the activity of alkaloids in insect science, the investigation methods issued in this kind of research can be of interest in medical research. Due to economical and ethical reasons, invertebrates, including insects, became important models in the first stage of drug designing.

The pharmacological ranges of concentrations and toxic levels are often close. Therefore, emphasis must be put on concentrations and doses, which may cause lethal and sublethal effects in mammals. This is important in the case of substances that are used in plant protection, food preservation, and hygiene of storage chambers and containers. From the human point of view, the toxic activity of substances, which are used as medicines, is equally, if not more important. Aconitum alkaloids are used in ethnomedicine and modern medicine, and their toxicity may be lethal for mammals. The data on the distribution of toxic alkaloids within the organs of the exposed individual is crucial for clinical toxicology [13]. In addition, some endophytes, like *Epichloe*, produce secondary metabolites that are toxic to insects. Therefore, they are potential sources of insecticides. Chanoclavine, an ergot alkaloid, was tested by Finch and co-workers against mice, to estimate their toxicity for a mammal model organism [14]. Although the mice revealed some neurotoxic symptoms, they were not permanent, and the median lethal dose was higher than 2000 mg per kg body weight. That suggested that the substance is relatively safe for mammals. However, further research is necessary, due to the reported toxicity of ergot alkaloids to mammals, including human. Additionally, the livestock that consumes ergot alkaloids shows various toxic symptoms, including endocrine disruption, reproductive and developmental malfunctions, and blood circulation [15]. The two review manuscripts present in this Special Issue proved the need for further extensive studies on the activity of alkaloids [11,15].

All the abovementioned studies proved the enormous potential of alkaloids in veterinary, pharmacology, medicine, and plant protection. Additionally, they showed multifold aspects of alkaloids and alkaloid-containing extracts toxicity from cytotoxicity through the malfunctions of organs and systems to lethal effects. Due to the increasing resistance of bacteria to antibiotics, they may become crucial for fighting microbial diseases. The description of postulated metabolic pathways influenced by the tested substances appeared to be very important for the planning of possible drugs in veterinary and medicine, as well as for basic science, like neurobiology or cell physiology. Similarly to bacteria developing resistance to antibiotics, insects develop resistance to insecticides. Hence, there is a need for new formulas, which may fight herbivore insects, with high selectivity against pests. Alkaloids are among the substances that are postulated as such novel insecticides. To sum up, the scientific and applicatory potential of alkaloids is immense. The research on their structure and activity develops intensively in various fields of science, which was proven by the variety of research topics present in this Special Issue. For sure, the number of research papers showing interesting and applicable pharmacological and toxicological aspects of alkaloids’ activity will be increasing.
